# Association of extreme hyperoxemic events and mortality in pediatric critical care: an observational cohort study

**DOI:** 10.3389/fped.2024.1429882

**Published:** 2024-07-31

**Authors:** Thomas E. Bachman, Christopher J. L. Newth, Patrick A. Ross, Nimesh Patel, Anoopindar Bhalla

**Affiliations:** ^1^ Department of Biomedical Technology, Faculty of Biomedical Engineering, Czech Technical University in Prague, Kladno, Czechia; ^2^ Department of Anesthesiology Critical Care Medicine, Children’s Hospital Los Angeles, University of Southern California Keck School of Medicine, Los Angeles, CA, United States; ^3^ Department of Anesthesiology, Vanderbilt University Medical Center, Nashville, TN, United States

**Keywords:** oximetry, hyperoxemia, mortality, pediatric critical care, oxygen toxicity

## Abstract

**Objective:**

Our aim was to confirm whether extreme hyperoxemic events had been associated with excess mortality in our diverse critical care population.

**Methods:**

Retrospective analysis of 9 years of data collected in the pediatric and cardiothoracic ICUs in Children's Hospital Los Angeles was performed. The analysis was limited to those mechanically ventilated for at least 24 h, with at least 1 arterial blood gas measurement. An extreme hyperoxemic event was defined as a PaO_2_ of ≥300 torr. Multivariable logistic regression was used to assess the association of extreme hyperoxemia events and mortality, adjusting for confounding variables. Selected a-priori, these were Pediatric Risk of Mortality III predicted mortality, general or cardiothoracic ICU, number of blood gas measurements, as well as an abnormal blood gas measurements (pH < 7.25, pH > 7.45, and PaO_2_ < 50 torr).

**Results:**

There were 4,003 admissions included with a predicted mortality of 7.1% and an actual mortality of 9.7%. Their care was associated with 75,129 blood gas measurements, in which abnormal measurements were common. With adjustments for these covariates, any hyperoxemic event was associated with excess mortality (*p* < 0.001). Excess mortality increased with multiple hyperoxemic events (*p* < 0.046). Additionally, treatment resulting in SpO_2_ > 98% markedly increased the risk of a hyperoxemic event.

**Conclusion:**

Retrospective analysis of critical care admissions showed that extreme hyperoxemic events were associated with higher mortality. Supplemental oxygen levels resulting in SpO_2_ > 98% should be avoided.

## Background

Mitigating exposure to hypoxemia is a fundamental necessity of critical care. Admission hypoxemia, but not hyperoxemia, has long been integrated into mortality prediction metrics such as PRISM, APACHE and SNAP. Admission hyperoxemia has been identified as a risk factor in pediatrics (1, 2). Further, concerns about avoiding hyperoxemia, except in neonatal care, have been studied but not widely adopted.

Clinicians have been aware of the profound effect of hyperoxemia on newborn infants for three-quarters of a century (3). Importantly the development and enhancement of oxygen measurement systems over the decades and a growing sensitivity to the risks associated with oxygen have resulted in better control and reduced the associated morbidity, most notably retinal damage. The pathogenesis of the inflammatory response is now well recognized. The balance between excess oxygen and inadequate oxygen in extremely premature neonates using oxygen saturation (SpO_2_) was evaluated in a coordinated series of large randomized controlled trials (4). They reported 5 years ago that decreased mortality in a lower saturation target range outweighed decreased morbidity in the higher target range. Nevertheless, the optimal neonatal target is still far from resolved (5–7).

Recent meta-analyses of dozens of randomized controlled studies evaluating the impact of liberal vs. conservative oxygenation in adult intensive care have not reached consensus as to whether conservative oxygenation has a mortality benefit (8, 9). Results from Oxy-PICU, a large multicenter trial evaluating liberal vs. conservative oxygenation based on SpO_2_ targets in 1,872 emergent admissions to the PICU, recently became available (10). They reported that conservative oxygen use, based on SpO_2_ target ranges, was associated with their primary outcome of organ failure free days. However, they found no mortality benefit. An associated subgroup analysis also showed a reduction in oxidative stress in the conservative oxygen group (11).

Hyperoxemia in children has been shown in observational studies to be associated with excess mortality, though most studies looked at only admission hyperoxemia or narrow populations (12). Recently an observational study of 5,406 children reported an increase in multisystem organ failure and mortality associated with hyperoxemia in the first day of admission (2). Further in a single site observational study of 6,250 PICU admissions, Ramgopal et al. reported that a single episode of extreme arterial oxygen (≥300 torr) during care was associated with excess mortality and, in addition, that multiple episodes were associated with increased excess mortality (13). They also reported an association of extreme arterial oxygen with extreme SpO_2_.

The aim of this retrospective observational study was to determine if the findings of Ramgopal et al. could be validated in our more diverse pediatric critical care population.

## Methods

### Design

This is a retrospective observational cohort study of an existing integrated patient and treatment information data repository at a single tertiary level children's hospital. Use of masked aggregated information from this medical records database was approved for this project by the institutional review board (approved 2/21/22 by CHLA-IRB, CHLA-23-0019, Implications of Hyperoxemia and Hypoxemia in ICU Patients). The study was conducted in compliance with the ethical standards of the IRB and the Helsinki Declaration of 1975.

### Population

The database of admissions to the cardiothoracic (CTICU) or general pediatric (PICU) critical care units between August 2012 and September 2021 was queried. Admissions with invasive mechanical ventilation and at least one arterial blood gas assessment were identified, and those with at least 24 h of mechanical ventilation were included in the analysis. Subjects that received extracorporeal membrane oxygenation (ECMO) were excluded.

### Analysis parameters

Demographics, gas exchange measurements, length of invasive mechanical ventilation, the predicted risk of mortality based on admission PRISM III score, and ICU mortality were gathered. Arterial blood gas measurements with PaO_2_ ≥ 300 torr associated with a brain death exam were excluded (<4 h before and <12 h after). Peripheral oxygen saturation measurements (SpO_2_) were extracted that coincided with the arterial blood gas measurements. The SpO_2_ value used was the mean of four 30-s values within ±1 min of the arterial sample.

### Endpoints

The highest PaO_2_ (max-PaO_2_) for each subject was determined, as descriptive measure of an episode of exposure to extreme hyperoxemia (13).

The primary outcome measure was ICU mortality in excess of expected mortality. Because we wished to compare our findings with a prior report (13), the primary independent parameter for extreme hyperoxemia was defined as one or more episodes of PaO_2_ ≥ 300 torr. A number of other independent potentially confounding covariates were selected a-priori. To adjust for admission risk of mortality the PRISM III probability of death was used. The incidence of other abnormal blood gas parameters during the course of the admission were specified as potentially confounding independent covariables. These included the presence or absence of a PaO_2 _< 50 torr, a pH < 7.25, and a pH > 7.45 during treatment. The number of arterial blood gas measurements [a measure of severity (13)] and ICU type (PICU, CTICU) were also included as independent covariates.

An extreme SpO_2_ was defined as 99%–100% (13, 14). Triggered by a recent publication of the Oxy-PICU trial (10), SpO_2_ strata reflecting liberal and conservative SpO_2_ management, were added. The aim of this analysis was to determine the association of individual PaO_2_ assessments within three SpO_2_ ranges (99%–100%, 94%–98%, 88%–92%). The first reflected the extreme of the SpO_2_ range and the latter two were chosen representative of the liberal and conservative oxygenation goals of the Oxy-PICU study. In addition to extreme hyperoxemia, we wished to also consider exposure to hypoxemia. An extreme hypoxemic event was defined as a PaO_2_ < 40 torr.

### Statistical analysis

Descriptive information was tabulated. A polynomial regression was constructed to describe the association of mortality in excess of the PRISM III prediction and max-PaO_2_. It included all max-PaO_2_ values, but is presented for the window between 50 and 500 torr. This was intended to depict the mortality differences associated with maximum arterial oxygen exposure.

Multivariable logistic regression was used for analysis to correct for a-priori identified potentially confounding covariates. Mortality was the dependent variable and extreme hyperoxemic (PaO_2_ ≥ 300 torr) event the primary independent variable. The 5 covariates are defined above. This final logistic regression model was also used to explore the sensitivity of increasing episodes of extreme hyperoxemia by replacing the dichotomous variable (any) with a trichotomous variable (0, 1, ≥2).

Univariate analyses were used to compare the differences in ICU populations (Mann-Whitney for continuous, and z-test for dichotomous variables). 95% confidence intervals were constructed to compare the mean likelihood of extreme hypoxemia and hyperoxemia at different SpO_2_ target strata.

A *p* < 0.05 was specified as statistically significant. Statistical tests were conducted with XLSTAT v11.5 (Lumivero, NY USA).

## Results

During the 9-year study period, there were 6,018 admissions requiring mechanical ventilation and arterial blood gas measurements. The analysis population, children receiving at least 24 h of mechanical ventilation and without ECMO exposure, was 4,003 separate admissions of 3,302 patients. During these admissions there were 75,129 arterial blood gas measurements, after excluding 246 associated with hyperoxemic brain death exams. There were a median of 12 (IQR 6–23) arterial blood gases measurements per admission. The median age was 7 months (IQR 1.0–73), and 57% were male. The median length of mechanical ventilation was 4.5 days (IQR 2.0–9.3). Among the 4,003 admissions, the average PaO_2_ during treatment was a median of 96 torr (IQR 66–125), while their max-PaO_2_ was a median of 149 torr (IQR 97–205). The predicted mortality (admission PRISM) was 7.1%. The actual mortality was 9.7%, 37% higher than mean predicted mortality. Sixty-three percent were in the CTICU and the balance in the PICU. As reported in Table 1, admissions in the CTICU were markedly younger, with lower levels of oxygenation, more prevalent PaO_2_ < 50 torr, as well as lower predicted and actual mortality. The incidence of PaO_2_ ≥ 300 torr, however, was slightly higher (11.7% vs. 9.4%).

**Table 1:** Analysis population by ICU.

	Total	PICU	CTICU	P (PICU-CTICU)
*n*	4,003	1,469	2,534	
Gender (% male)	57%	55%	58%	ns
Age (months)	7.0 (1.0–73)	89 (22–171)	2 (0–8)	<0.001
Mechanical ventilation (days)	4.5 (2.0–9.3)	5.2 (2.7–11)	4.0 (1.9–8.3)	<0.001
max-PaO_2_ (torr)	149 (97–205	158 (121–200)	139 (75–210)	<0.001
Average PaO_2_ (torr)	96 (66–125)	107 (87–131)	85 (50–118)	<0.001
Average SpO_2_ (%)	98 (93–99)	99 (97–100)	97 (86–99)	<0.001
Average FiO_2_ (%)	40 (34–53)	37 (32–47)	42 (35–55)	<0.001
Average PaCO_2_ (torr)	43 (39–46)	41 (38–46)	43 (40–46)	<0.001
Average pH	7.40 (7.37–7.42)	7.39 (7.36–7.42)	7.40 (7.38–7.42)	<0.001
predicted mortality (%)	7.1	11.5	4.5	<0.001
Actual mortality (%)	9.7	19.6	4.0	<0.001
# arterial blood gas	12 (6–23)	9 (4–21)	13 (7–25)	<0.001
Any PaO_2 _≥ 300 (%)	10.8	9.4	11.7	0.021
Any PaO_2 _< 50 (%)	39	16	51	<0.001
Any pH < 7.25 (%)	21	30	16	<0.001
Any pH > 7.45 (%)	65	57	70	<0.001

Values presented as median(IQR) or % proportion. Predicted mortality based on admission PRISM III. Average value reflects the median of the subjects’ values.

**Figure 1:**
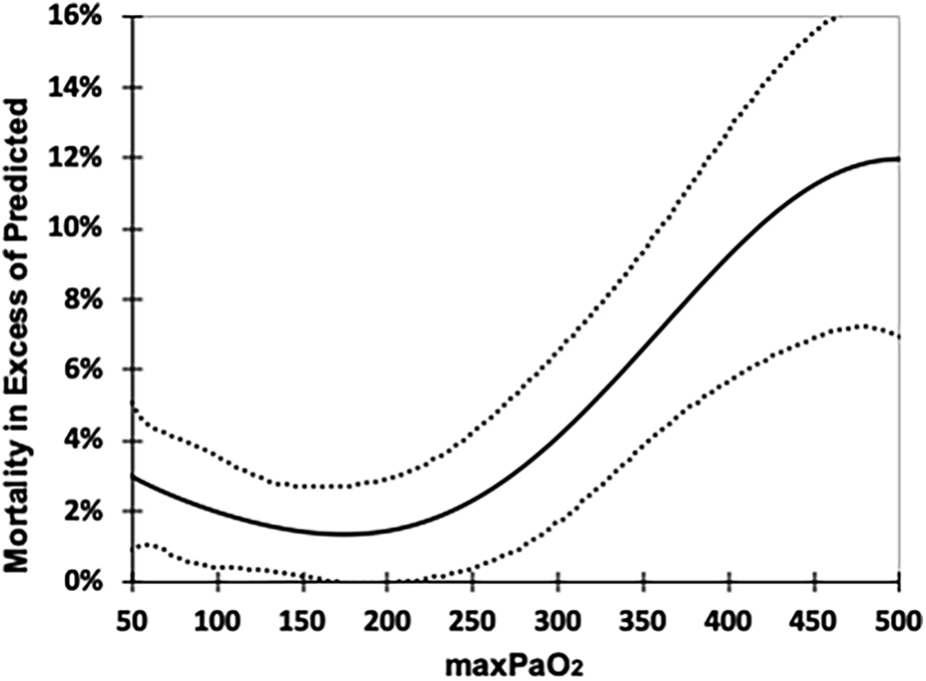
Excess mortality associated with max-PaO_2_. Excess mortality is calculated as the Actual Mortality minus the % predicted mortality (PRISM III), 6th order polynomial fit. Dotted lines 95% CI. Regression reflects all values (14–642 torr) but range of chart truncated for clarity (50–500 torr).

Figure 1 is a best fit polynomial plot of the actual mortality in excess of the PRISM III predicted mortality across the range of max-PaO_2_. There is a clear association of max-PaO_2_ with excess mortality (*p* < 0.001) and the relationship is *J*-shaped, with increased excess mortality associated with persistent hypoxemia and hyperoxemia.

The results of the multivariable analysis reporting the association of mortality adjusting for the independent covariates is shown in Table 2. The presence of any extreme hyperoxemic events (PaO_2_ ≥ 300 torr) in 10.8% of the subjects was statistically significantly associated with mortality, {[2.39 OR (95% 1.69–3.38) *p* < 0.001]}. All the covariates were also statistically associated with mortality. The relative size of their effect is proportional to the odds ratio. Of note are the relative impact of other abnormal blood gas measurements. The presence of a pH < 7.25 markedly increased the likelihood of excess mortality, while a pH > 7.45 decreased it. The impact of the former was greater than a hyperoxemic event. A PaO_2_ < 50 torr was associated with a similar impact of a hyperoxemic event.

**Table 2:** Logistic regression: covariable association with mortality.

Independent covariable	Prevalence	Odds ratio (95% CI)	*P*
*n*	4,003	Na	Na
Any PaO_2_ ≥ 300 (%)	10.8	2.39 (1.69–3.38)	<0.001
Probability of death (%)	7.1	1.04 (1.03–1.04)	<0.001
Number of ABGs	12 (6–23)	1.01 (1.01–1.02)	<0.001
Any PaO_2 _< 50 (%)	39	1.73 (1.25–2.04)	0.001
Any pH < 7.25 (%)	21	5.29 (3.98–7.04)	<0.001
Any pH > 7.45 (%)	65	0.63 (0.47–0.85)	0.043
PICU (%)	37	5.78 (4.17–8.00)	<0.001

Values presented as median(IQR) or % proportion. Predicted mortality based on admission PRISM III.

Additionally, when the presence of any extreme hyperoxemic event (PaO_2 _≥ 300 torr) was replaced with increasing number of hyperoxemic events, ≥2 events was associated with increased mortality [OR 1.89 (1.01–3.53) *p* = 0.045] compared to 1 event (Supplementary Table S1). Finally, as a sensitivity analysis, the multivariate logistic regression was repeated separately for the CTICU and PICU admissions, and an extreme hyperoxemic event remained statistically associated with excess mortality in each (*p* < 0.001).

Assessment of the association of extreme hyperoxemic events with SpO_2_, in the subset of all 75,125 gas exchange observations is shown in Table 3. Of these observations, 67% were in the 3 defined SpO_2_ strata (99%–100%, 94%–98%, 88%–92%). The former representing hyperoxemic levels and the other two liberal and conservative SpO_2_ targets. The results are shown for the CTICU and PICU separately. The likelihood of a PaO_2_ ≥ 300 torr in both the conservative and liberal SpO_2_ targets was markedly lower than SpO_2_ of 99%–100%. There was, however, a marked decrease (less than half) in the likelihood of a PaO_2_ ≥ 300 torr in the conservative targeting strata compared to the liberal strata. However, there was also a marked increase (more than triple) in the likelihood of an extreme hypoxemic event (PaO_2_ < 40 torr) in the liberal targeting strata. These were evident for both ICUs, though the absolute levels were different.

**Table 3:** Likelihood of extreme PaO_2_ by SpO_2_ Strata.

SpO_2_ strata	99%–100% hyperoxemia	94%–98% liberal	88%–92% conservative
*N*			
CTICU	15,272	10,390	4,224
PICU	11,374	7,501	1,683
PaO_2_ ≥ 300 (% likelihood)			
CTICU	2.9 (2.6–3.2)	1.5 (1.2–1.7)	0.4 (0.2–0.6)
PICU	1.8 (0.5–2.0)	0.7 (0.5–0.9)	0.3 (0.0–0.6)
PaO_2_ < 40 (% likelihood)			
CTICU	2.8 (2.6–3.1)	4.3 (3.9–4.7)	16 (15–17)
PICU	0.4 (0.3–0.5)	0.4 (0.3–0.6)	1.7 (1.1–2.3)

Values are likelihood (95% CI).

## Discussion

We identified an observational cohort of 4,003 pediatric critical care admissions requiring mechanical ventilation in our ICUs. We used a multivariate analysis, controlling for covariates, to evaluate the association of extreme hyperoxemic events (PaO_2_ ≥ 300) and excess mortality. We found that extreme hyperoxemic events during the course of treatment were independently associated with excess mortality. To our knowledge this is the first confirmation of Ramgopal's report (13). Further, we were also able to demonstrate that excess mortality associated with hyperoxemic events holds true for cardiothoracic ICU admissions.

We showed that the relationship of excess mortality and highest-PaO_2_ was *j-shaped*. The nadir of the lowest excess mortality was among those with a max-PaO_2_ of 150–200 torr. The excess mortality of those subjects whose max-PaO_2_ was above 250–300 torr exceeded those with persistent hypoxemia (max-PaO_2_ < 50 torr). In addition, the magnitude of excess mortality increased as max-PaO_2_ increased above 300 torr. These findings are consistent with Ramgopal (13), who reported excess mortality associated with max-PaO_2_ < 100 torr, and max-PaO_2_ ≥ 300 torr. Further our analysis suggests that SpO_2_ levels of 99%–100% should be avoided entirely, as they double the likelihood of a PaO_2_ ≥ 300 torr compared to an SpO_2_ 94%–98%.

Our findings associating blood gas covariates with mortality are interesting and warrant further prospective research. It appears that exposure to pH levels below 7.25 were associated with marked excess mortality while those above 7.45 with reduced mortality. Further, hypoxemic exposure (PaO_2_ < 50 torr) was also associated with increased mortality. The latter is important in that SpO_2_ is available continuously, and thus exposure to hypoxemia and hyperoxemia can be continuously monitored and managed.

Our cohort differed somewhat from the Ramgopal cohort, that is it focused on sicker admissions, and including cardiothoracic diagnoses. We feel this provides more robust support to the association between extreme hyperoxemia and excess mortality. Regardless of these differences in population, both studies found a similar highly significant independent increase in mortality associated with extreme hyperoxemic events. However, these findings are seemingly in contrast to the recently published Oxy-PICU randomized controlled trial, and the most recent meta-analysis of adult trials.

Oxy-PICU found no mortality benefit of conservative oxygen control, but reduced severity of organ failure (10). There are two points to consider. First there is an important difference in the aim of these studies that merit consideration. Our study and that of Ramgopal evaluated whether a prospectively defined extreme hyperoxemic event (PaO_2_ ≥ 300 torr) was associated with excess mortality. In contrast Oxy-PICU evaluated whether prescribing a lower target range (88%–92% SpO_2_) improved outcome compared to a usual liberal oxygenation strategy (SpO_2 _> 94). Second, our analysis of the association of SpO_2_ with extreme arterial hypoxemia and hyperoxemia at different SpO_2_ target ranges is helpful in understanding the apparent contrast regarding mortality. Our data showed that maintenance of the conservative target range would markedly reduce the chance of a PaO_2_ ≥ 300 torr, and thus, assuming an effect, reduce mortality. However, our data also found that the lower conservative SpO_2_ target range would be associated with a marked increase in extreme hypoxemic events, that would likely increase mortality. This is consistent with recent report, from a small cohort of the Oxy-PICU subjects, which found higher markers of hypoxia in the conservative group (11). Thus, the lower conservative target range may have been too low. These same issues of uncertainty of optimal SpO_2_ target ranges persist in neonatology (5–7). Further, both the liberal and conservative management cohorts had ample exposure to SpO_2_ between 99%–100%, thought it was lower in the conservative arm. Finally, we note that an extreme episode of hyperoxemia can occur regardless of the target range and adequacy of compliance.

Unfortunately, there is not a definitive answer available when looking at data in critically ill adults. The impact of oxygenation control in adult critical care has been thoroughly evaluated, without clear consensus after dozens of studies. In a 2018 meta-analysis of 25 studies, Chu et al. concluded that high quality evidence supports conservative use of supplemental oxygen, reporting a 20% reduction in hospital mortality (9). A 2023 Cochrane Collaboration report evaluated of 17 studies concluded there was no mortality benefit associated with a conservative oxygen strategy, but a possible reduction in severe adverse events (8). This evaluation also superseded an earlier Cochrane review of 7 studies concluding that conservative oxygenation may have mortality benefit (15). The order of magnitude of the reduction of mortality in the Chu and the first Cochrane analyses (9, 15) was similar. The differences in the timing of the mortality endpoint, highlighted in the recent Cochrane report (8), does not rule out consistency among all three.

We suggest, consistent with others (10, 11, 16, 17) that extreme arterial hyperoxemic events may lead to significant oxidative stress in critically ill subjects and subsequent risk of excess mortality. However, we cannot rule out that extreme hyperoxemic events are indicative of an over-response to significant hypoxemic events, which by circumstance might not be captured with a coincident blood gas. This would suggest that hyperoxemic events are really a marker of cardiopulmonary instability and severe hypoxemic events. We further suggest that periodic blood gases cannot be used to manage an oxygenation target. Rather, continuous SpO_2_ is essential in responding to hypoxemia and avoiding high levels of SpO_2_ to reduce the likelihood of extreme hyperoxemia.

Several additional limitations should be taken into account when considering the applicability of our results. First, we could not exclude subjects with uncorrected cardiac anomalies at both admission and discharge due to the granularity of diagnostic codes. However, we estimate there were few. Second an observational study can only identify associations not demonstrate cause and effect. Regardless, a strength of the study is that the primary independent factor (PaO_2_ ≥ 300 torr) and other analysis covariables were identified *a-priori*. Nevertheless, different cutoffs for extreme hyperoxemia and different covariables might have resulted in different conclusions and warrant further evaluation. Finally, this is an analysis of a wide variety of etiologies over nearly a decade, and while it is reflective of our patient population, more focused investigations are merited.

## Conclusions

We confirmed that in our diverse population including general and cardiothoracic infants and children, extreme hyperoxemic arterial levels of PaO_2_ ≥ 300 torr are independently associated with excess mortality. We suggest that extreme hyperoxemic events should be avoided, most likely by severely limiting exposure to SpO_2_ readings of 99%–100%.

## Data Availability

The data analyzed in this study is subject to the following licenses/restrictions: data available upon reasonable request. Requests to access these datasets should be directed to CN, cnewth@chla.usc.edu.
