# Supplementary material for: Association of extreme hyperoxemic events and mortality in pediatric critical care: an observational cohort study
Source: Front Pediatr. 2024 Jul 31;12:1429882. doi: 10.3389/fped.2024.1429882 (PMC11322569; doi:10.3389/fped.2024.1429882)
Supplement: Supplementary file 1 [file Table1.pdf]

Supplementary Table 1

Independent variables	Incidence	Odds Ratio (95% CI)	P
n	4003	na	na
0 vs ≥2 PaO_2 ≥300 (%)	89%/4.7%	3.34 (2.07-5.41)	<0.001
1 vs ≥2 PaO_2 ≥300 (%)	4.7%/6.1%	1.89 (1.01-2.44)	0.046
Probability of death	7.1%	1.03 (1.03-1.04)	<0.001
Number of ABGS	12 (6-23)	1.01 (1.01-1.02)	<0.001
Any PaO_2<50 (%)	39%	1.76 (1.27-2.44)	0.001
Any pH<7.25 (%)	21%	5.34 (4.03-7.09)	<0.001
Any pH>7.45 (%)	65%	0.62 (0.46-0.84)	0.002
PICU (%)	37%	5.95 (4.29-8.26)	<0.001

sTable 1 Table 3 Covariate association with Mortality with categorical hyperoxemic events The categories are 0 (none), 1 event and 2 or greater events.
